# Construction and analysis of cardiac hypertrophy-associated lncRNA-mRNA network based on competitive endogenous RNA reveal functional lncRNAs in cardiac hypertrophy

**DOI:** 10.18632/oncotarget.7312

**Published:** 2016-02-10

**Authors:** Chao Song, Jian Zhang, Yan Liu, Hao Pan, Han-ping Qi, Yong-gang Cao, Jian-mei Zhao, Shang Li, Jing Guo, Hong-li Sun, Chun-quan Li

**Affiliations:** ^1^ Department of Pharmacology, Harbin Medical University-Daqing, Daqing, China; ^2^ Department of Medical Informatics, Harbin Medical University-Daqing, Daqing, China

**Keywords:** cardiac hypertrophy, lncRNA, ceRNA, gene expression profile, network analysis, Pathology Section

## Abstract

Cardiac hypertrophy (CH) could increase cardiac after-load and lead to heart failure. Recent studies have suggested that long non-coding RNA (lncRNA) played a crucial role in the process of the cardiac hypertrophy, such as Mhrt, TERMINATOR. Some studies have further found a new interacting mechanism, competitive endogenous RNA (ceRNA), of which lncRNA could interact with micro-RNAs (miRNA) and indirectly interact with mRNAs through competing interactions. However, the mechanism of ceRNA regulated by lncRNA in the CH remained unclear. In our study, we generated a global triple network containing mRNA, miRNA and lncRNA, and extracted a CH related lncRNA-mRNA network (CHLMN) through integrating the data from starbase, miRanda database and gene expression profile. Based on the ceRNA mechanism, we analyzed the characters of CHLMN and found that 3 lncRNAs (SLC26A4-AS1, RP11-344E13.3 and MAGI1-IT1) were high related to CH. We further performed cluster module analysis and random walk with restart for the CHLMN, finally 14 lncRNAs had been discovered as the potential CH related disease genes. Our results showed that lncRNA played an important role in the CH and could shed new light to the understanding underlying mechanisms of the CH.

## INTRODUCTION

Cardiac hypertrophy (CH) was a slowly compensatory function of heart due to pressure overload for a long time and was considered as the main cause of heart failure [1]. CH was the result of cardiac muscle non-normal enlargement originating from an increase in cell size of cardiomyocytes. Though it was an initially adaptive response to normalize wall tension and maintain cardiac output, various experimental and clinical studies had verified that continuous CH was a rather maladaptive process, ultimately leading to heart failure [1, 2]. Based on the previous studies, coding RNAs and non-coding RNAs were high related to CH [3]. Although non-coding RNAs couldn't code the proteins, they could exert their functions by regulating the coding-RNAs. Recent years, as a class of the non-coding RNA, long non-coding RNA (lncRNA) had attracted wide attention because of the abundant and crucial function in complex biological regulation. They exerted their function by regulating translation, splicing and gene expression [4, 5]. What's more, lncRNA was related to the occurrence and development of many diseases [6].

Now research had shown that some lncRNAs played an important role in the development of cardiovascular disease [7]. LncRNA caused the dysfunction of normal heart and eventually led to the disease by indirectly regulating the downstream genes' expression [8]. For instance, Kurian et al. discovered 3 novel lncRNAs (TERMINATOR, ALIEN and PUNISHER) that regulated the cardiovascular development processes [9], and Han et al. firstly found the lncRNA Mhrt inhibited cardiac hypertrophy and failure by reducing the expression of Brahma-related gene 1 (Brg1) [10]. However, there were only a few lncRNAs that had been verified by the low through-put experiments related to the CH. High through-put experiments (microarray, RNA-seq) could detect many lncRNAs by one time experiment. So the high through-put experiments data and bioinformatics methods were preformed to predict the potential CH disease lncRNAs, which had been considered viable ways.

**Figure 1:**
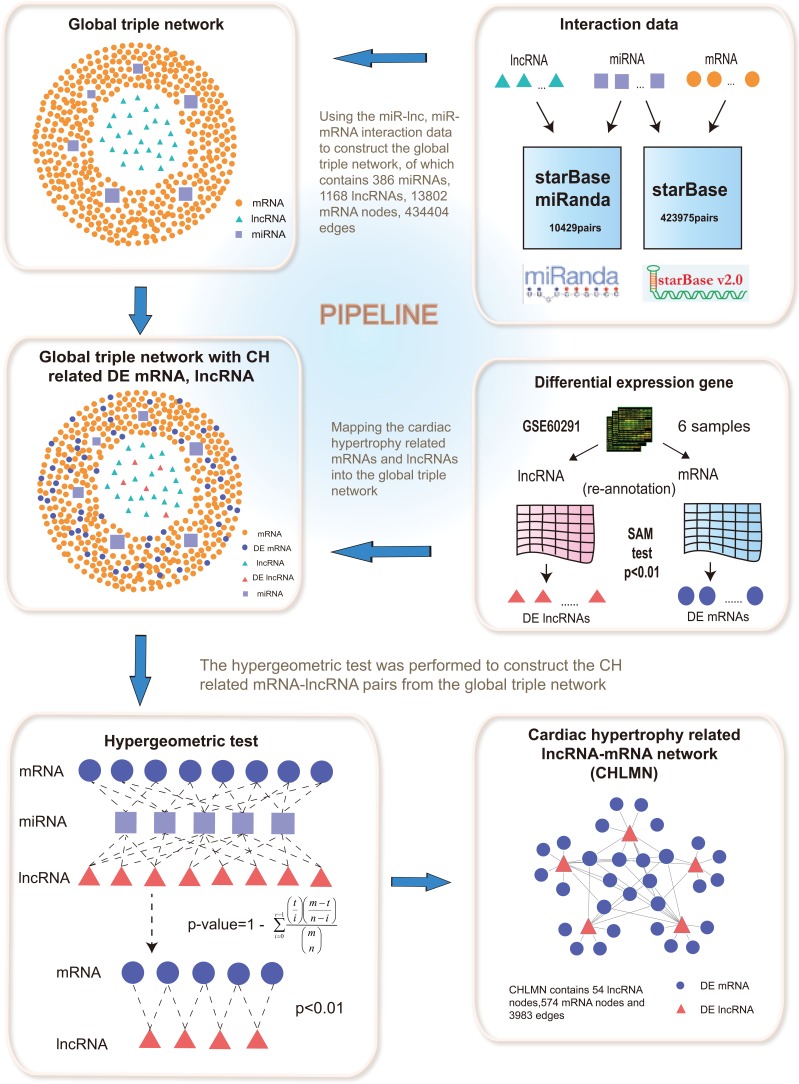
An integrative pipeline for the construction of global triple network and CHLMN Interactions between lncRNAs and miRNAs were provided from starbase database and miRanda prediction tools. The interactions between miRNAs and mRNAs were provided from starbase. The global triple network was constructed by merging all the interactions of lncRNA-miRNA, miRNA-mRNA. Mapping the CH related differential expressed lncRNAs and mRNAs (computed from gene expression profile) into the global triple network. If differential expressed lncRNAs and mRNAs shared the same miRNAs could add an edge between them, using hypergeometric test, then the CHLMN was constructed.

Competitive endogenous RNA (ceRNA) was a novel regulatory mechanism between non-coding RNA and coding RNA, that had been proposed recently [11]. LncRNA competitively interacted with the miRNA, thus inhibited mRNA degradation that the same miRNA targeted [12]. Salmena et al. had found the ceRNA interaction from the cancer [11]. However, the ceRNA mechanism related to CH is unclear. In our study, based on the ceRNA theory, we constructed a global triple network by using the data from starbase database and miRanda algorithm, in the light of lncRNA and mRNA from a triple sharing the same miRNA. We extracted the cardiac hypertrophy related lncRNA-mRNA network (CHLMN) by processing the gene microarray data to obtain the differentially expressed genes, and mapped the differentially expressed lncRNAs and mRNAs into the global triple network. The pipeline of CHLMN's construction had been shown in Figure 1. We then used the CHLMN to: (1) investigate different topological properties of the network and miner the lncRNA modules, (2) implement bidirectional hierarchical cluster analysis of the CHLMN, (3) perform random walking with restart to the CHLMN. We identified CH related lncRNAs with the high reliability, and our results showed that lncRNA played an important role in the CH and could shed new light to the understanding underlying mechanisms of the CH.

## RESULTS

### Identification of CH associated differential expressed lncRNAs and genes based on probe re-annotation

As a result of the data shortage of the lncRNA microarry annotation data in the GEO database, some studies had shown that lncRNA annotation data could be exploited by the re-annotation methods, so we developed a measure for re-annotation and performed it to annotate the lncRNA and mRNA annotation sets (see in method). Firstly, we downloaded one open gene expression profile GSE60291 of CH with 6 samples (3 CH, 3 control), which probe sequences were supported by Affymetrix Human genome U133 plus 2.0 array. Sequence alignment algorithm was applied between probe annotation sequences and human long non-coding transcript sequences, protein-coding transcript sequences from Gencode database, respectively, using the Blastn tools. Then through the three-level quality filtering, we identified two (lncRNA-probe, mRNA-probe pairs) annotation sets, respectively. There were 14620 mRNA-probes, 4184 lncRNA-probes pairs we got totally, and we represented these genes in Ensembl ID.

According to the re-annotation results, we calculated the 6 samples by the means of SAM test (see in method) to identify the differential expressed genes. We considered *p*-value < 0.01 was statistically significant. We identified 791 differential expressed mRNAs and 222 differential expressed lncRNAs, respectively. Because of the coherence of the data, we converted the gene Ensembl ID to gene symbol through the biomart id converters (http://www.biomart.org/). Finally, on account of the data loss of different databases in the process of converting gene id, we got 789 differential expressed mRNAs and 64 differential expressed lncRNAs. These differential expression results were seen as potential key regulators in the occurrence and development of the CH.

### Construction of CH related lncRNA-mRNA network from global triple network

**Figure 2:**
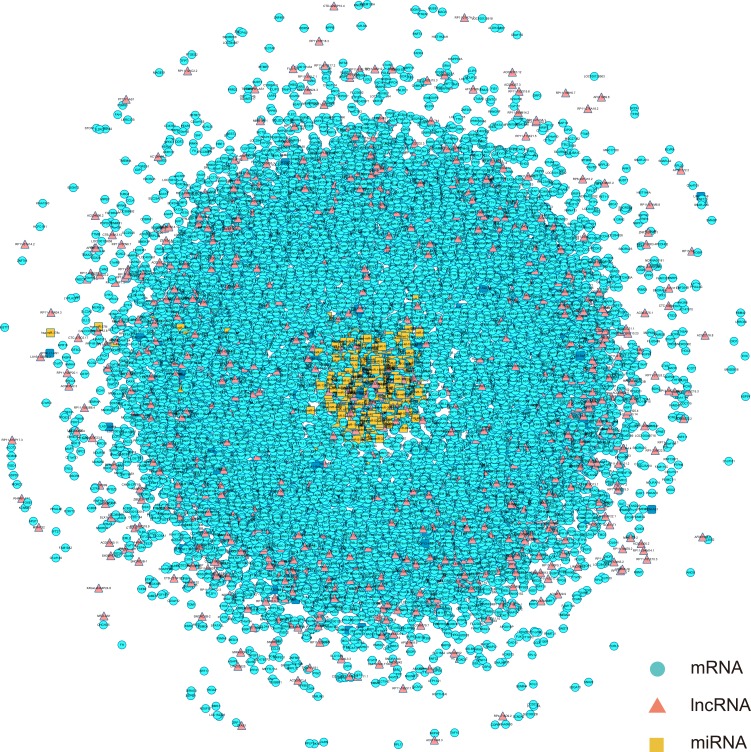
The view of global triple network The blue nodes represented mRNA, the red nodes represented lncRNAs, and the yellow nodes represented miRNAs. There were 386 miRNAs, 1168 lncRNAs, 13802 mRNAs and 434404 edges in the network.

To construct global triple network, we downloaded the human miRNA-mRNA interactions from the starbase V2.0 database. In total, we obtained 423975 miRNA-mRNA interaction pairs. For the miRNA-lncRNA interactions, the data came from two databases, starbase and miRanda. We downloaded the human miRNA-lncRNA interactions from the starbase V2.0 database, then we preformed the miRanda tools to predict the lncRNAs appeared in previous 64 differential expressed lncRNAs that could interact with the known miRNAs in the starbase database. We downloaded human miRNA transcript sequence from Gencode database and searched for the differential expressed lncRNA's sequences from UCSC genome browser. The threshold we set was prediction score > 160 and energy < −20. We considered the threshold was the perfect match. In total, we obtained 10429 miRNA-lncRNA interaction pairs. Finally, we merged all miRNA-mRNA pairs and miRNA-lncRNA pairs to construct global triple network. We defined the miRNA, mRNA and lncRNA as the nodes of the network. If they interacted each other, we added an edge between them, then the global triple network had been constructed (Figure 2).

**Figure 3:**
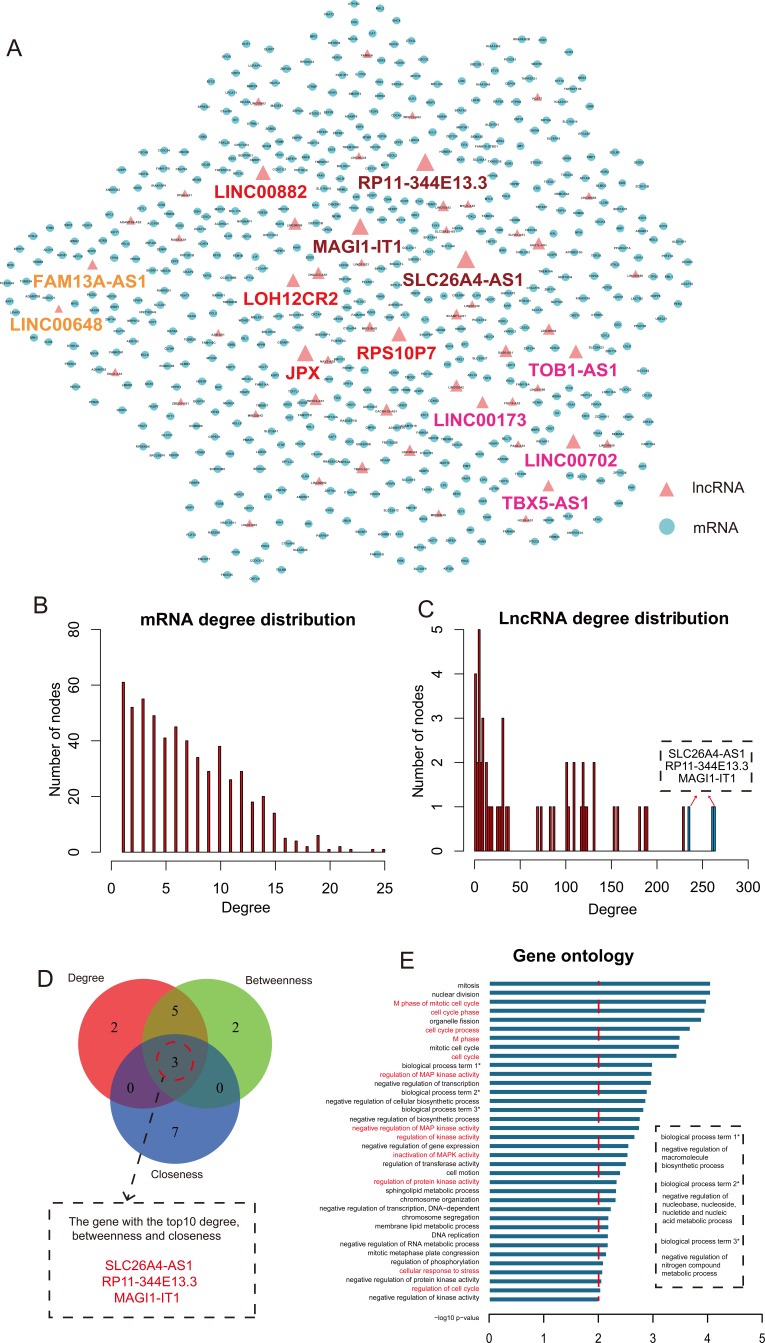
The topological features of CHLMN **A.** The view of CHLMN. The blue nodes represented CH related differential expressed mRNAs and the red nodes represented CH related differential expressed lncRNAs, the nodes with colorful label were the lncRNAs that high related to CH in our study and the same color represented they came from one analysis. The size of nodes represented the degrees of the nodes in the network. **B.** The node distribution of mRNA. X-axis was the degrees of the nodes and the y-axis were the number of nodes. **C.** The node distribution of lncRNA. X-axis were the degrees of the nodes and the y-axis were the number of nodes. The bars in blue were the top 3 max degree lncRNA nodes in the network. **D.** The Venn diagram of the top 10 max nodes in each dimension (degree, betweenness and closeness). **E.** The gene ontology (GO) enrichment analysis of the lncRNA RP11-344E13.3, and x-axis were the −log10 of *p*-value. We thought *p* < 0.01 was the statistical significant. Y-axis was the names of biological processes, the whole name of the label with * showed in the dotted box and the names with red were the CH high related biological processes.

In order to observe the relationship of the triplet in the CH and identify the CH related lncRNAs, we mapped 789 CH-associated differential expressed mRNAs and 64 CH-associated differential expressed lncRNAs into global triple network (Figure 2). The result showed that there are 624 mRNA and 56 lncRNA mapped into the global triple network. We extracted these genes and their linked miRNAs to a new triple pairs, we then performed the significance test of the number of shared miRNAs between lncRNA and mRNA pairs using hypergeometric test (see in method). If a mRNA and a lncRNA shared the same miRNA and significance score with *p*-value <0.01, then added an edge between them. We finally constructed a new CH related lncRNA-mRNA network (Figure 3A). In the network, there were 574 mRNA nodes, 54 lncRNA nodes and 3983 edges (Figure 3A).

### Topological analysis of CH related lncRNA-mRNA network

**Figure 4:**
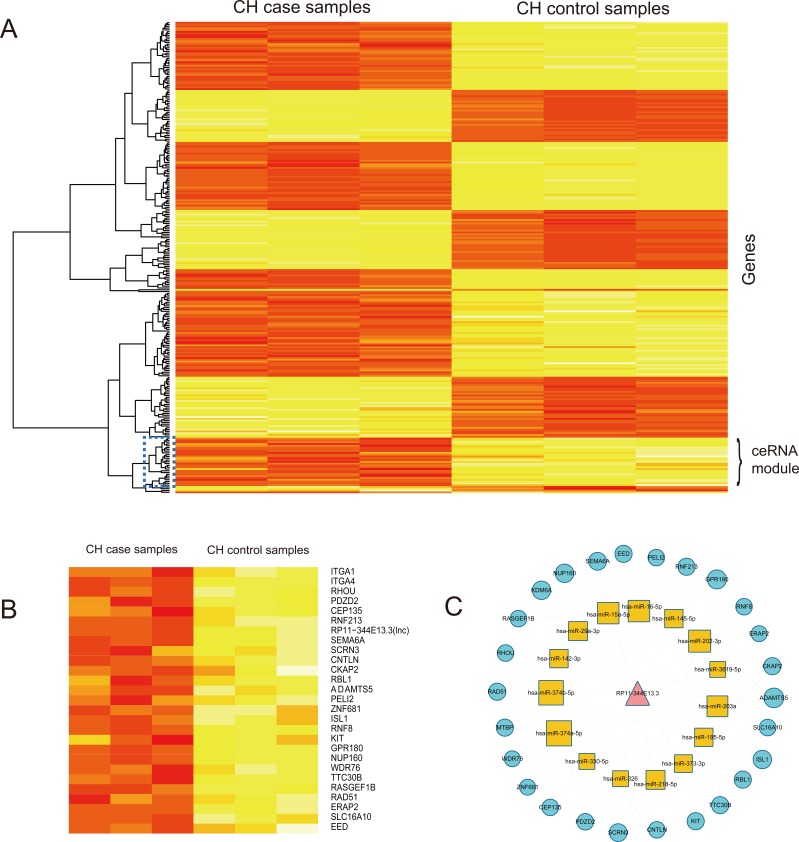
Cluster analysis of lncRNA RP11-344E13.3 and their mRNA neighbors in CHLMN **A.** The heatmap of lncRNA RP11-344E13.3 and their mRNA neighbors based on their expression. The columns represented 6 samples and the rows represented lncRNA and its mRNA neighbors. The dark blue rectangle in the map represented the co-expression ceRNA module. **B.** The detailed ceRNA module heatmap extrated from (A), labels on the right were the names of lncRNA and mRNAs. **C.** The ceRNA module network of lncRNA RP11-344E13.3 extracted from global triple network.

We firstly analyzed the topological properties of the CHLMN. We calculated the degree, closeness and betweenness of the CHLMN, respectively. The mRNA and lncRNA node distribution had shown in Figure 3B and 3C. We ranked all the nodes' topological features of the network. We listed the top 10 of each dimension (Table 1). Interestingly, we found that 3 lncRNAs (SLC26A4-AS1, RP11-344E13.3, MAGI1-IT1) appeared in each of the list (Figure 3D). For the further explanation, we performed gene ontology (GO) function and KEGG pathway annotations for each of the three lncRNAs by using their first mRNAs neighbors in CHLMN (Supplementary Table S1-S6). For the lncRNA RP11-344E13.3, we found that the biological processes contained “regulation of MAP kinase activity”, “cellular response to oxidative stress”, “inactivation of MAPK activity” and the KEGG pathways enriched included “ECM-receptor interaction”, “Hypertrophic cardiomyopathy (HCM)” (Figure 3E). Many studies had shown that these biological processes and pathways were closely related to CH. MAPK signal pathways consisted of lots of kinases that ultimately result in the dual phosphorylation and activation of p38, JNKs and ERKs [13]. The MAPK signalling cascade was initiated in cardiac myocytes by GPCRs, receptor tyrosine kinases (IGF-I, and fibro blast growth factor receptors), receptor serine/threonine kinases (transforming growth factor-β (TGF-β)), cardiotrophin-1 (gp130 receptor), and by stress stimuli such as stretch [14]. Activated p38, JNKs and ERKs each phosphorylate multiple intracellular targets, including numerous transcription factors that induced the reprogramming of cardiac gene expression and cardiac hypertrophy [15]. Thum et al. had found that the ERK-MAP kinase signalling pathway in cardiac fibroblasts had impacts on global cardiac structure and function. In a pressure-overload-induced disease model reduced cardiac ERK-MAP kinase activity, inhibited interstitial fibrosis and attenuated cardiac dysfunction [16]. In the biological processe of “regulation of MAP kinase activity”, we enriched the gene “PAK1”, the previous study had shown that the PAK1 activation was able to attenuate hypertrophy and offered a novel therapeutic strategy for the CH [17]. Muthuramu et.al. had found that selective reducing the oxidative stress could affect cardiac remodeling and function in a model of pressure overload-induced cardiomyopathy induced by transverse aortic constriction [18]. In addition, Hong et.al found that ECM-receptors interaction may be involved in the cardioprotective effects of curcumin, which had extensive cardioprotective effects against cardiac hypertrophy and myocardial infarction [19]. The other two lncRNAs were performed the same annotation, the biology processes and KEGG pathways enriched were highly related to the CH, too, such as “MAPKKK cascade” and “ECM-receptor interaction”. These results suggested that the three lncRNAs with higher degree, betweenness and closeness were important in the network and played a crucial role in the origin and development of the CH. At the same time, we performed cluster analysis to the expressions of each of the three lncRNA and their first mRNA neighbors in CHLMN (Supplementary Figures S1, S2). For the lncRNA RP11-344E13.3, the heatmap result had showed in the Figure 4A. Interestingly, we could find that a handful of mRNAs had clustered with the lncRNA (the region showed in the Figure 4B). According to the previous knowledge, we had known that the lncRNA and mRNA appeared to the co-expression patterns in the ceRNA. So we extracted the handful of mRNAs, lncRNA and extracted their linked miRNA in the global triple network, constructing new ceRNA module network by using these genes (Figure 4C). In the ceRNA module network, we could find that some miRNAs were related to CH by searching for literatures. Huang et.al had found that attenuation of miR-16 could repress expression of cyclins D1, D2 and E1, and activate cyclin/Rb pathway to induce the cardiomyocyte hypertrophy, revealing that miR-16 might be a target to manage cardiac hypertrophy [20]. Tijsen et.al had found that miR-15 family could target to TGF-βR1 and several other genes in the TGF-β pathway, including P38, SMAD3, SMAD7 and endoglin, which result in inhibiting the TGF-β-pathway in the heart [21]. Some studies had found that miR-145 could target to CaMKIIδ to suppress ROS-induced Ca^2+^ overload of cardiomyocytes and target to GATA6 to inhibits isoproterenol-induced cardiomyocyte hypertrophy [22, 23]. As for the other two lncRNAs, we also found that the media miRNAs of the triplet were high related to CH (data shown in the Supplementary Table S9, S10, S11 and S12), suggesting that the three lncRNAs with higher degree, betweenness and closeness played an important role in the CH.

**Table 1:** The top 10 genes in degree, betweenness and closeness

Gene	degree	Gene	betweenness	Gene	closeness
**SLC26A4-AS1**	264	**SLC26A4-AS1**	33646.19	**SLC26A4-AS1**	0.000768
**RP11-344E13.3**	262	**RP11-344E13.3**	27888.12	**RP11-344E13.3**	0.000763
**MAGI1-IT1**	235	**JPX**	16777.8	**ITGB8**	0.000755
**JPX**	230	**MAGI1-IT1**	16025.51	**SEMA6D**	0.000751
**RPS10P7**	190	**LINC00702**	14569.72	**ARID5B**	0.000742
**LINC00882**	188	**ASH1L-AS1**	11119.95	**LMNB1**	0.000736
**LINC00702**	181	**TOB1-AS1**	10599.61	**GLCCI1**	0.000736
**TOB1-AS1**	158	**GAS6-AS1**	10363.3	**ZNF367**	0.000735
**LOH12CR2**	154	**LINC00882**	10248.51	**MAGI1-IT1**	0.000734
**LINC00086**	132	**RPS10P7**	8653.479	**DYRK1A**	0.000732

There were 3 lncRNAs (SLC26A4-AS1, RP11-344E13.3 and MAGI1-IT1) appeared in each dimension. The node with higher degree, betweenness and closeness played crucial roles in the network.

### Module analysis of CH related lncRNA-miRNA network

**Figure 5:**
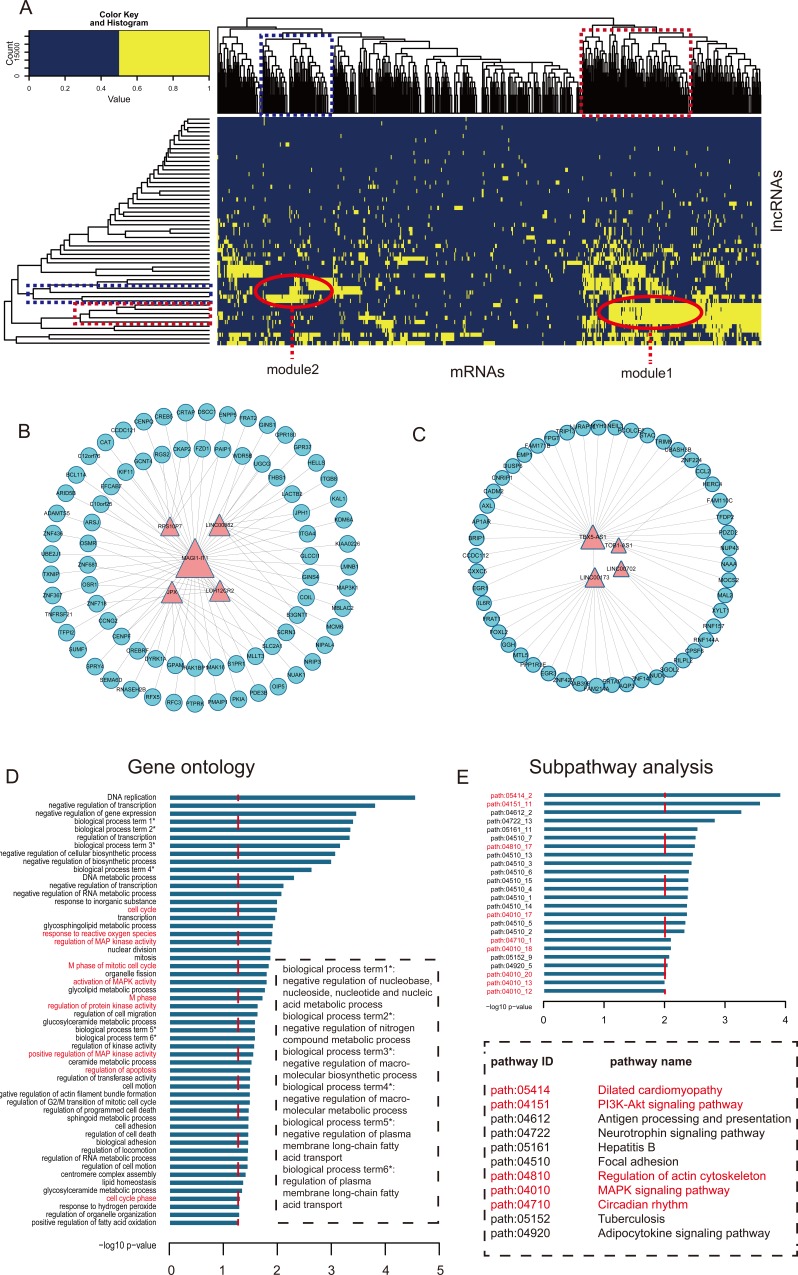
Module analysis of the interaction of lncRNAs and mRNAs **A.** The heat map of CHLMN. The bidirectional hierarchical cluster was performed by R package. The two red ellipses represented module 1 and module 2, respectively (The same for the rectangles in the cluster tree). **B.** Module 1 network extracted from CHLMN. **C.** Module 2 network extracted from CHLMN. **D.** The gene ontology (GO) enrichment analysis of module 1, x-axis were the −log10 of *p*-value and we thought p < 0.05 was the statistical significant. Y-axis was the names of biological processes, the whole name of the label with * showed in the dotted box and the names with red were the CH high related biological processes. **E.** Subpathway enrichment analysis of the module 1, x-axis were the −log10 of *p*-value and we thought p < 0.01 was the statistical significant. Y-axis was the names of subpathway, the pathway names were showed in the dotted box and the names with red were the CH high related subpathways.

**Figure 6:**
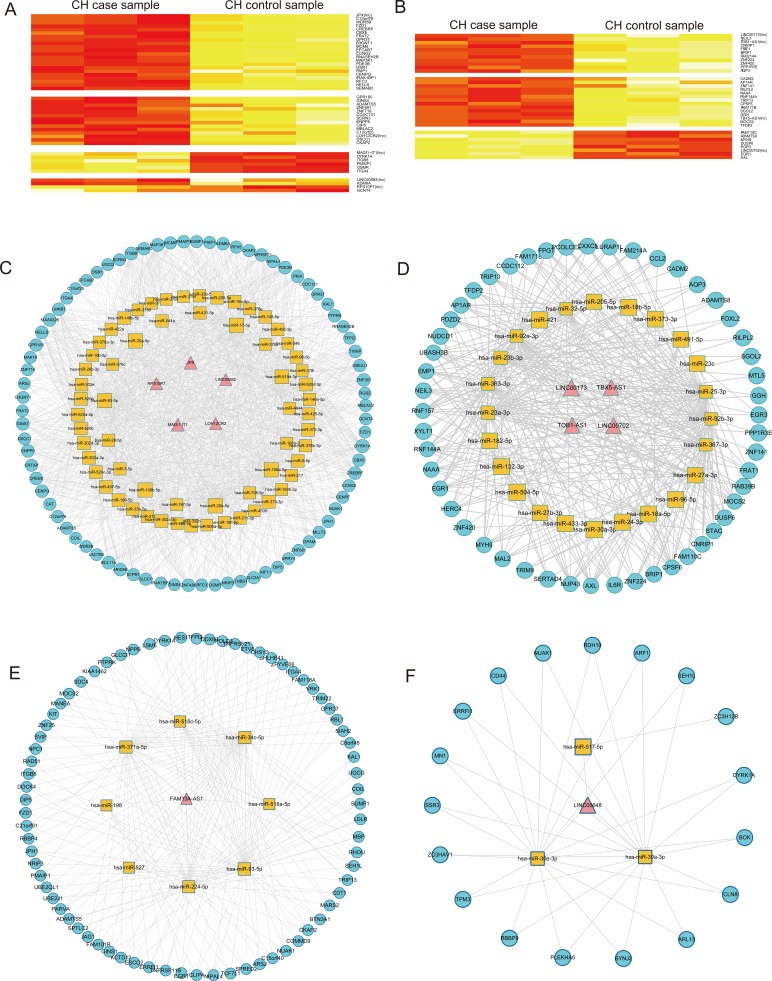
Analysis of CH-associated lncRNAs from ceRNA and random walk **A.** The heatmap of lncRNAs and mRNAs in the module 1 based on expression. The columns represented 6 samples and the rows represented lncRNAs and their mRNA neighbors. Labels on the right were the names of lncRNAs and mRNAs. **B.** The heatmap of lncRNAs and mRNAs in the module 2 based on expression data. The columns represented 6 samples and the rows represented lncRNAs and their mRNA neighbors. Labels on the right were the names of lncRNAs and mRNAs. **C.** The ceRNA module network of module 1 extracted from global triple network. **D.** The ceRNA module network of module 2 extracted from global triple network. **F.** The triple network of lncRNA FAM13A-AS1 extracted from global triple network. **E.** The triple network of lncRNA LINC00648 extracted from global triple network.

To further investigate the cross-talks between mRNAs and lncRNAs, we performed bidirectional hierarchical clustering on the CHLMN by using R package “gplots”. In the heat map (Figure 5A), we discovered 2 modules (Figure 5B and 5C) were highly related to CH. In module 1, we performed GO enrichment analysis and subpathway enrichment analysis for these genes in the module (Figure 5D and 5E). The result showed that “regulation MAP kinase activity”, “regulation of apoptosis” and “cell cycle” were significant and high related to the CH. In the above text, we had explained that the crucial role of MAP kinase in the development of the CH. A significant decrease in phosphorylation of p44/p42 MAP kinases (ERK) was detected in the AAC-treatment model rat [24], and a group had verified that the three map kinases (ERK, JNK, P38) are significant changed in the external stress stimuli through high-throughput experiment [25]. Apoptosis was the process of programmed cell death (PCD) that may occur in multicellular organisms. Some studies had confirmed that the activity of apoptosis was participated in the development of CH. Apoptosis of cardiomyocytes played an important role in the transition from cardiac hypertrophy to heart failure. Hypertrophied cardiomyocytes showed enhanced susceptibility to apoptosis [26]. The enrichment results of module 2 were also high related to CH, the data had shown in the Supplementary Table S7 and S8. Just like the network topology analysis, we performed cluster analysis to the expressions of the two modules (Supplementary Figures S3 and S4). Based on the ceRNA, we also found ceRNA modules with CH related co-expression lncRNAs and mRNA each in module 1 and module 2 (Figure 6A and 6B), and we extracted their media miRNAs in the global triple network to construct a new network (Figure 6C and 6D). In module 1 ceRNA network (Figure 6C), we found some miRNAs were related to CH according to other group's studies. Shehadeh et.al had found that microRNA-20a constrains p300-driven myocardial angiogenic transcription by direct targeting of p300 and miR-20a was demonstrated to directly repress p300 expression through a consensus binding site in the p300 3′UTR [27]. Interestingly, in the module ceRNA network, we found that there are many mir-378 family miRNAs. Some studies had found that miR-378 family played a crucial role in the development of the CH. Deficiency of cardiomyocyte-specific microRNA-378 contributed to the development of cardiac fibrosis involving a transforming growth factor β (TGF-β_1_)-dependent paracrine mechanism [28]. MiR-378, as a regulator of cardiomyocyte hypertrophy, exerted its activity by suppressing the MAPK signaling pathway on several distinct levels [29]. Deficiency of miR-378 alone was sufficient to induce fetal gene expression, which was prevented by knocking down Grb2 expression and blocking Ras activation, thus suggesting that miR-378 interferes with Ras activation by targeting Grb2, and Ras signaling was related to the CH [30]. In module 2 ceRNA network (Figure 6D), we also found that some miRNAs were highly related to the CH. Above all, the two modules were highly related to CH, and according the two clustering strategies, we could find the crucial CH related lncRNAs in the local dimension.

### Random walk analysis of CH related lncRNA-mRNA network

For the CHLMN, we selected five known disease related mRNA nodes (“PPARGC1A”, “CPT1A”, “LPL”, “NPPB”, “TNFRSF11B”) from the CHLMN as the seed nodes, the method of random walk (see in methods) with restart was performed to prioritize CH high related lncRNAs. We found two lncRNAs (FAM13A-AS1, LINC00648) were statistical significant (*p*-value<0.05) after 3000 times random walk with permutation (the data had shown in the Supplementary Table S9). As we known that the lncRNA with high significant scores suggested that it connected the seed nodes in their close neighbors and their functions were high corrected with the seed nodes. So we considered the two lncRNAs were the potential regulators of the CH. We extracted their mRNA neighbors in the CHLMN, the miRNAs between these lncRNAs and mRNAs to construct two triple networks (Figure 6E, 6F). Refer to the miRNAs in the two triple network, Bernardo et.al have demonstrated that inhibition of the miR-34 family, but not miR-34a alone, provided benefit in a chronic model of myocardial infarction and inhibition of miR-34a in mice with moderate cardiac pathology attenuated atrial enlargement and maintained cardiac function [31]. Pan et.al had found that miR-30a induced alterations in beclin-1 gene expression and autophagy in cardiomyocytes, angiotensin II induces down-regulation of miR-30 in cardiomyocytes and circulating miR-30 may be an important marker for the diagnosis of left ventricular hypertrophy [32]. Duistersm et.al had indicated that miR-30 importantly limit the production of Connective tissue growth factor (CTGF), a key molecule in the process of fibrosis and therefore seems an attractive therapeutic target of cardiac hypertrophy [33]. Thus it could be seen the two lncRNAs' crucial roles in the CH.

## DISCUSSION

In our study, we used the interactions data from starbase database and miRanda algorithm to generate a global triple network based on the theory of ceRNA that the lncRNA and mRNA shared the same miRNA in one triplet. From the global triple network, we extracted the cardiac hypertrophy related lncRNA-mRNA network (CHLMN) by mapping the differential expressed genes into the global triple network for the further detail analysis of lncRNA's function. The CHLMN contained 574 mRNA nodes, 54 lncRNA nodes and 3983 edges, we implemented the topological properties and cluster analysis to the CHLMN, finally, we performed the random walk with restart to the CHLMN, according to these analysis, we discovered 14 lncRNAs were high related to the development of CH. Of which, the lncRNA RP11-344E13.3, connecting many mRNAs that were high related to the CH by GO enrichment analysis and pathway analysis, and the lncRNA could directly interact with a number of miRNAs, that were verified are the important factors in the development of CH. For instance, the miR-16 and miR-15 family could target the disease genes to participate in the CH, based on the ceRNA theory, maybe exist a triplet that contained RP11-344E13.3, miR-16 or miR15 and the downstream CH related disease mRNAs, the lncRNA could target with the miRNA and decrease the degradation of mRNA. Our method could deeply miner a lot of the potential triplets.

Our methods also exists some limitations. First of all, in the field of CH, there were not enough data to use. We downloaded the CH related profile GSE60291 that contained 6 samples (3 control, 3 case), and the SAM test was performed to calculate the differential expressed genes. Because of the lack of sample, maybe the result exited false positive. Secondly, microarray often focused more on testing the mRNAs expression, so less lncRNA were found through probe re-annotation on microarray. And if probe re-annotation were used in exon microarray, more lncRNAs might be annotated. Finally, in the process of converting gene different IDs from different databases, because of the disunity of database, a great deal of genes were lost, this may decrease our result. If exited a multi-samples data, our result would be more improved.

In our study, we efficiently identified 14 lncRNAs correlated to CH on the strength of ceRNA theory. We generated a new strategy to do research on CH or other disease. At the same time, the lncRNA played an important role in the CH and could offer new therapeutic targets for CH mechanism research.

## MATERIALS AND METHODS

### miRNA-mRNA interaction data

The interaction of miRNA-mRNA in our study was obtained from the starbase V2.0 database. Starbase was designed for decoding interaction networks of lncRNAs, miRNAs, competing endogenous RNAs (ceRNAs), RNA-binding proteins (RBPs) and mRNAs from large-scale CLIP-Seq (HITS-CLIP, PAR-CLIP, iCLIP, CLASH) data [34]. We downloaded 423975 miRNA-mRNA interaction pairs, including 13802 mRNAs, 1127 lncRNAs and 386 miRNAs.

The interaction of miRNA-lncRNA in our study was obtained from the starbase V2.0 database and miRanda prediction tools. We downloaded the human miRNA-lncRNA interaction data from starbase database and got 10212 miRNA-lncRNA interaction pairs. We also applied the miRanda tools for predicting the potential miRNAs that these differential expression lncRNAs targeted. miRanda was an algorithm for finding genomic targets for microRNAs. Combining the two data sources, we got 10442 miRNA-lncRNA interaction pairs.

In total, we got 423975 miR-mRNA interaction pairs and 10429 miRNA-lncRNA interaction pairs, including 386 miRNAs, 1168 lncRNAs and 13802 mRNAs.

### Gene expression profile

We downloaded the CH related gene expression profile from the GEO database (http://www.ncbi.nlm.nih.gov/geo/) under accession number of GSE60291 [35]. GEO was a public functional genomics data repository supporting MIAME-compliant data submissions. Array- and sequence-based data were accepted. Tools were provided to help users query and download experiments and curated gene expression profiles. There were 6 samples (3 control and 3 case), and the case were stimulated by ET-1 in cardiomyocytes [35]. ET-1 could cause the hypertension, increase the after-load, and result in the cardiac hypertrophy consequently.

### Probe re-annotation

The probe annotation sequences supported by the Affymetrix (http://www.affymetrix.com/) were aligned to the human long non-coding transcript sequences and human protein-coding transcript sequences from the GENCODE database (http://www.gencodegenes.org/) by performing BLASTn tools, respectively. Results from the sequence alignment were filtered as follows:

1. Only reserved the probes matched to one transcript, removed the probes matched to the protein-coding transcripts and long non-coding transcripts, resulting in two sets of probes-transcripts pairs, respectively.
2. In each set of probes-transcripts pairs, removed the probes matched to more than one transcript.
3. Each transcript should be perfectly matched to more than three probes.

### Identify the differential expression gene

Significance analysis of microarrays (SAM) [36] was applied to analyze the two phenotype (normal and cardiac hypertrophy disease) samples to discover differential expression genes, respectively. SAM test was based on the t-test and adjusted according to the characteristic that the noise of microarray data was correlated with the peak value of expression data. In this method, we considered that *p*-value < 0.01 was statistically significant.

### Hypergeometric test

We extracted CHLMN from global triple network by performing hypergeometric test. We considered *p*-value < 0.01 was statistically significant. The *p*-value was measured as:

$$\text{P}=1-\sum_{i=0}^{r-1}\frac{\left(\frac{t}{i}\right)\left(\frac{m-t}{n-i}\right)}{\left(\frac{m}{n}\right)}$$

where,

m represented total number of human genome miRNA
t represented number of miRNA interacting with the mRNA
n represented number of miRNA interacting with the lncRNA
r represented number of miRNA shared between mRNA and lncRNA

### Random walk with restart to prioritize CH related lncRNAs

A random walk in network was defined as an iterative walker's transition from its a certain node to a randomly selected neighbor that started from a given node (e. g. “Gene 1” was a known gene). In our study, the random walk we performed had capacity of restart with probability r in every time step at node “Gene 1”. The random walk with restart was defined as:

$${\text{p}}^{\text{t}+1}=(1-\text{r}){\text{Wp}}^{\text{t}}+{\text{rp}}^{0}$$

where W represented the column-normalized adjacency matrix of the network, p^t^ was a vector that size is equivalent to the number of nodes in the network and the i-th element holds the probability of being at node i at time step t.

In our study, the initial probability vector p^0^ was constructed such that 1 was assigned to the nodes representing known genes associated with disease, and other nodes with 0. We considered that the role of genes related to disease was equivalent in network. Vector p would be in the steady-state at time step t where t approached infinity as a limit. The iteration would be finished till the change between p^t^ and p^t+1^ fell below 10^−10^.

The random walk algorithm was performed in CHLMN to prioritize lncRNAs related to CH, and we performed statistical significance analysis for the scores of each lncRNA. The statistical significance for rejection of the null hypothesis was determined by comparing the scores of lncRNAs in the network following n iterations of that known CH related genes shuffling. In order to strictly keep the network topological properties, random sampling without replacement was performed when doing the random disturbance, and the degree distribution was guaranteed the same between selection seed node and the real. In iterations the times that the score of every lncRNA was higher than the real one was record as m. The statistical significance *p*-value for every lncRNA was calculated by the ratio of m and n. In this work, n was set at 3,000 times.

### Topological feature

The degree of a vertex was the number of edges that link to a given node. K was the number of edges that linked to node i. It could be defined as:

Deg (i)=K

For a given graph, G= (V,E) with n nodes, the betweenness B_i_ could be defined as:

$${\text{B}}_{\text{i}}=\frac{1}{\left(n-1)(n-2\right)}\sum_{s\neq i\neq t}\frac{s_{st}(i)}{s_{st}}$$

Where, S_st_ represented the number of shortest paths from node s to node t, and S_st_ (i) represented the number of shortest paths from node s to node t that passed through the node i.

Closeness (C_i_) represented how close a node was to all others in the same network and was defined as the average mean path from a node to all other nodes reachable from it:

$${\text{C}}_{\text{i}}=\frac{1}{\sum_{j-1}^{n}d(i,j)}$$

Where d (i, j) represented the shortest distance between the node i and the node j, and n represented the number of nodes of the network.

### Enrichment analysis

To realize functional enrichment, we performed DAVID 6.7 tools (https://david.ncifcrf.gov/) for gene ontology (GO) enrichment. DAVID provided a comprehensive set of functional annotation tools for investigators to understand biological meaning behind large list of genes [37]. After GO functional enrichment analysis, we considered the biology process terms with *p*-value < 0.01 or *p*-value < 0.05 was statistically significant.

KEGG (Kyoto Encyclopedia of Genes and Genomes, http://www.genome.jp/kegg/) was a database resource for understanding high-level functions and utilities of the biological system, such as the cell, the organism and the ecosystem, from molecular-level information, especially large-scale molecular datasets generated by genome sequencing and other high-throughput experimental technologies. Genes were enriched in the KEGG pathways by applying the iSubpathwayMiner package [38]. This package adopted the k-clique concept to define a subpathway. All possible subpathways were obtained by searching all possible paths between start-points and end-points in the adjacency matrix generated by node relationships in an entire KEGG pathway. The k was set to 4 in this study, which means that the distance among all genes within the subpathways was no greater than 4. We considered the subpathway with *p*-value <0.01 was statistically significant

## SUPPLEMENTARY FIGURES AND TABLES




















