# Supplementary material for: Construction and analysis of cardiac hypertrophy-associated lncRNA-mRNA network based on competitive endogenous RNA reveal functional lncRNAs in cardiac hypertrophy
Source: Oncotarget. 2016 Feb 10;7(10):10827–40. doi: 10.18632/oncotarget.7312 (PMC4905442; doi:10.18632/oncotarget.7312)
Supplement: Supplementary file 1 [file oncotarget-07-10827-s001.pdf]

Construction and analysis of cardiac hypertrophy-associated lncRNA-mRNA network based on competitive endogenous RNA reveal functional lncRNAs in cardiac hypertrophy

Supplementary materials

Table S1: The result of gene ontology analysis of SLC26A4-AS1. We used its mRNA neighbors in CHLMN and we thoght p-value < 0.01 was statistical significant.

Table S2: The result of KEGG pathway analysis of SLC26A4-AS1. We used its mRNA neighbors in CHLMN and we thought p-value < 0.05 was statistical significant.

Table S3: The result of gene ontology analysis of RP11-344E13.3. We used its mRNA neighbors in CHLMN and we thought p-value < 0.01 was statistical significant.

Table S4: The result of KEGG pathway analysis of RP11-344E13.3. We used its mRNA neighbors in CHLMN and we thought p-value < 0.05 was statistical significant.

Table S5: The result of gene ontology analysis of MAGI1-IT1. We used its mRNA neighbors in CHLMN and we thought p-value < 0.01 was statistical significant.

Table S6: The result of KEGG pathway analysis of MAGI1-IT1. We used its mRNA neighbors in CHLMN and we thought p-value < 0.05 was statistical significant.

Table S7: The result of gene ontology analysis of module 2. We used the lncRNAs' mRNA neighbors in CHLMN and we thought p-value < 0.05 was statistical significant.

Table S8: The result of subpathway analysis of module 2. We used the lncRNAs' mRNA neighbors in CHLMN and we thought p-value < 0.01 was statistical significant.

Table S9: The result of random walk of CHLMN. We selected 5 seeds (disease genes) to perform random walk and identified 2 lncRNAs high related to CH.

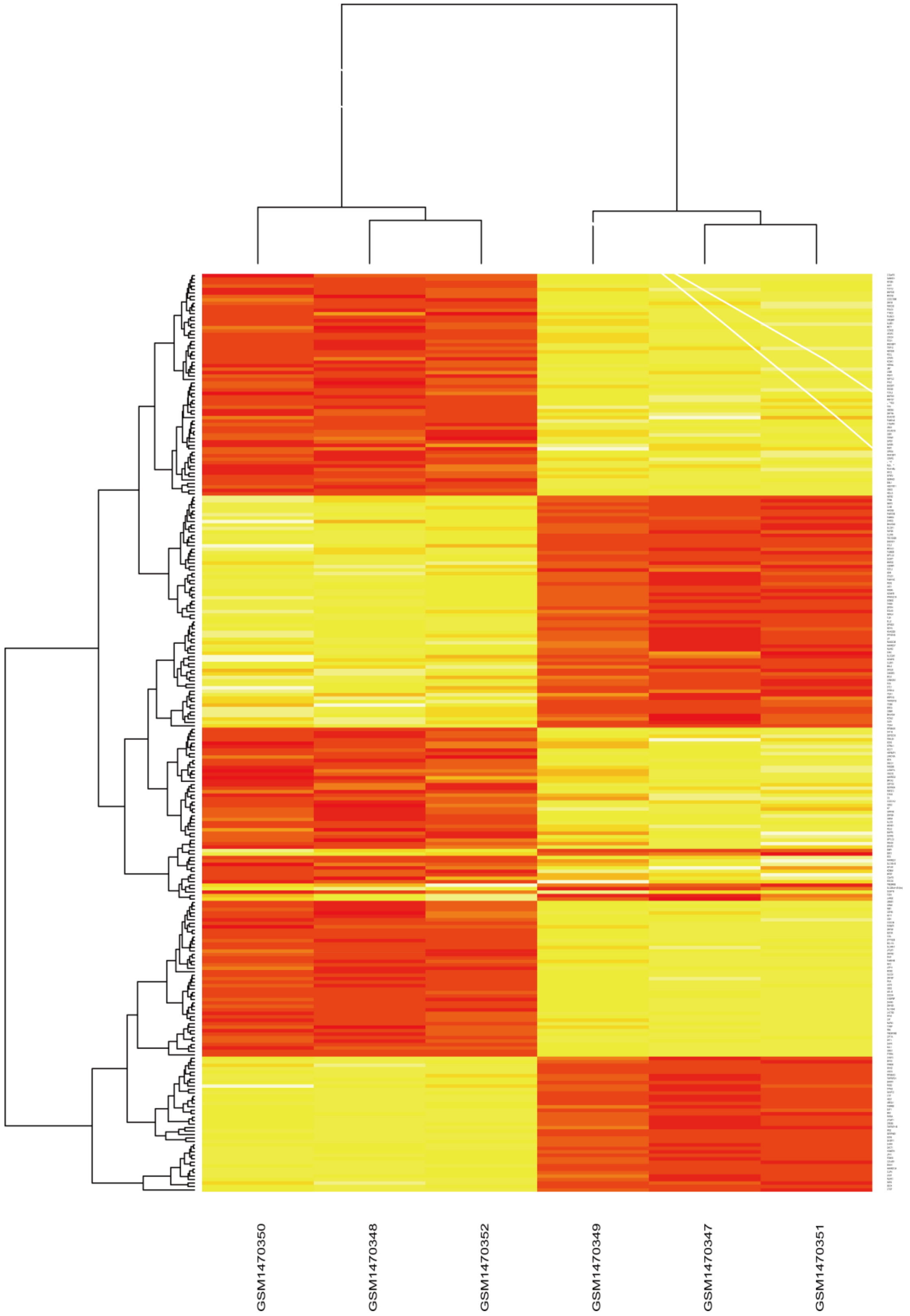

Figure S1: The heatmap of lncRNA SLC26A4-AS1 and their mRNA neighbors based on expression data. The columns represented 6 samples and the rows represented lncRNA and its mRNA neighbors.

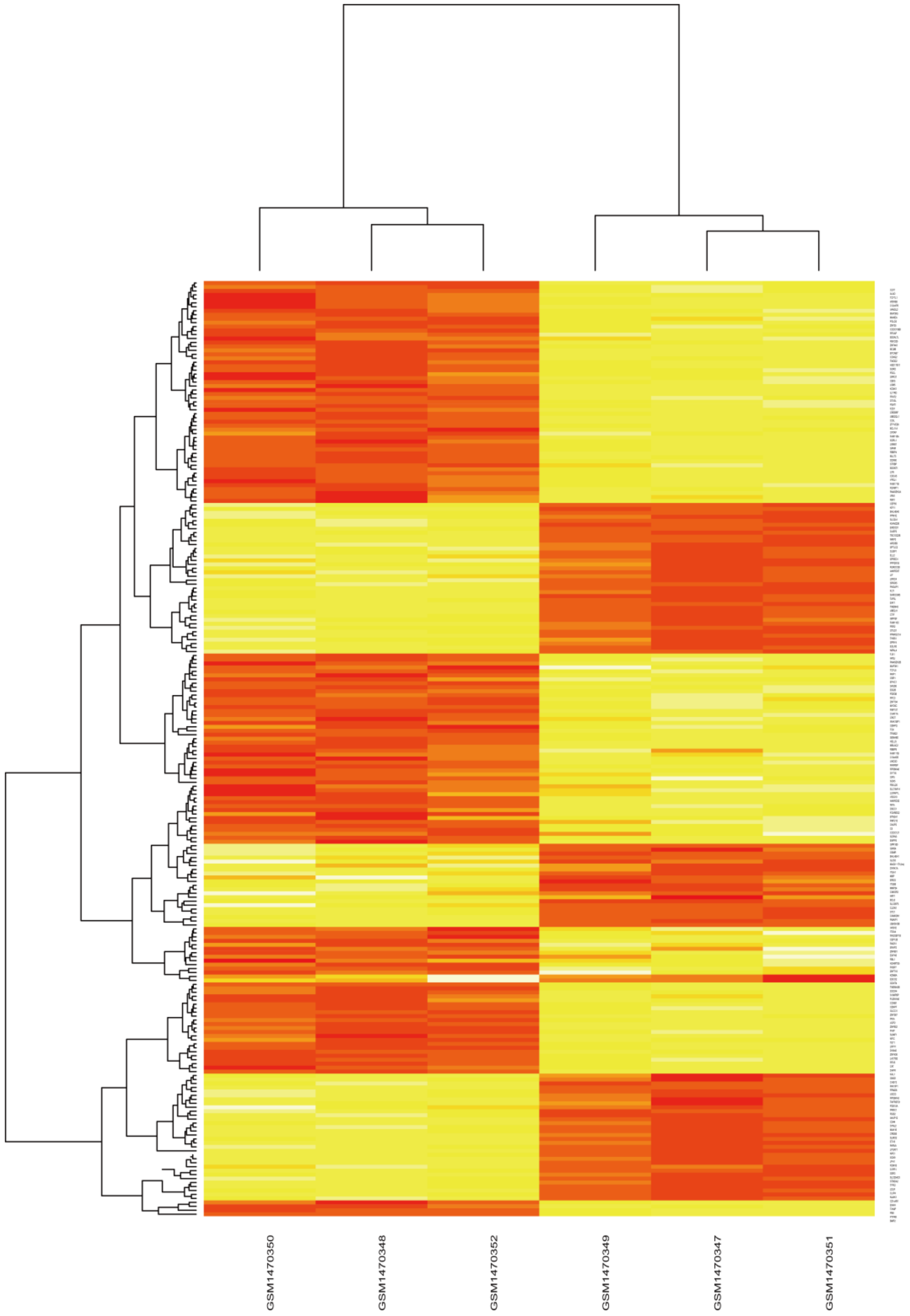

Figure S2: The heatmap of lncRNA MAGI1-IT1 and their mRNA neighbors based on their expression. The columns represented 6 samples and the rows representedof lncRNA and its mRNA neighbors.

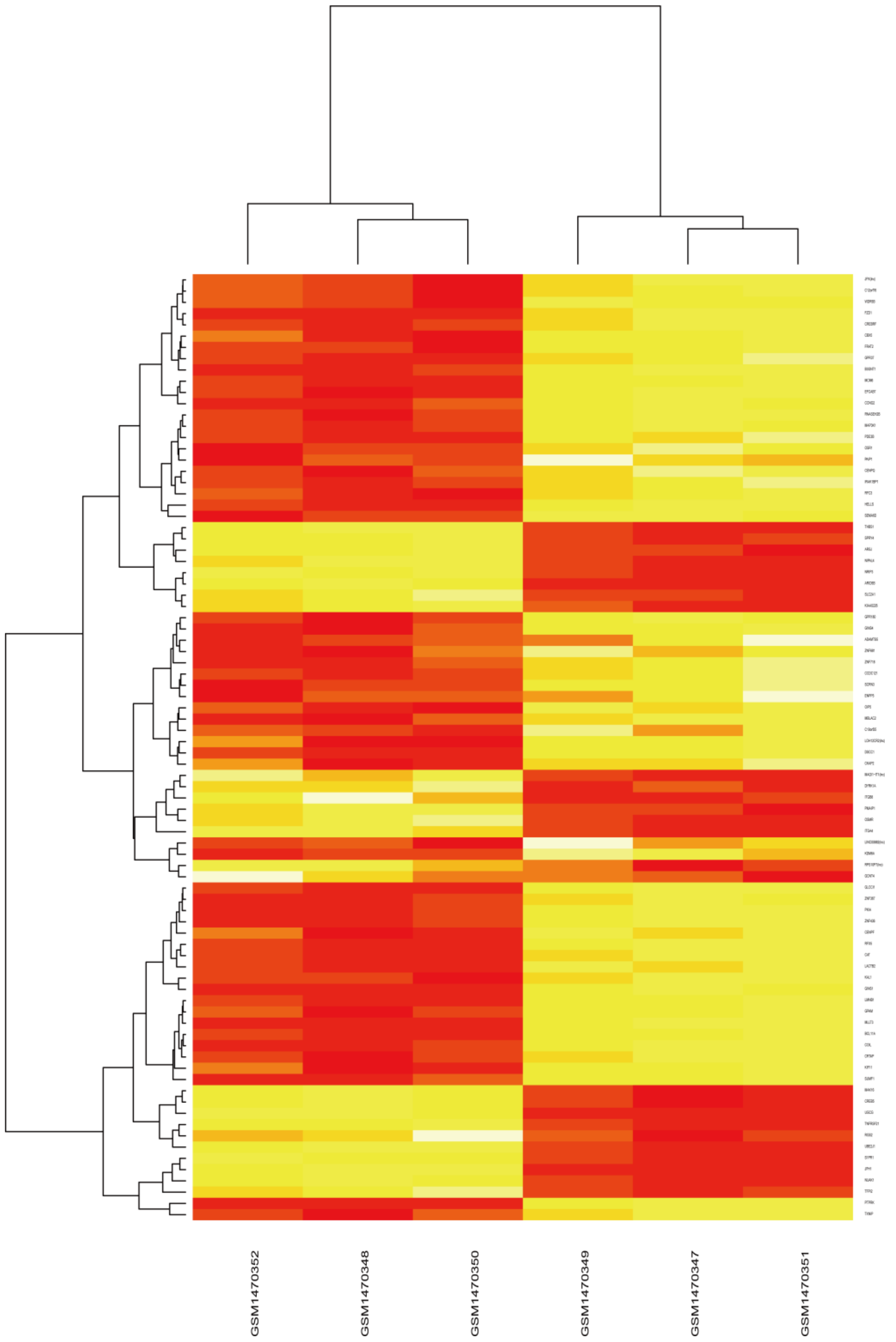

Figure S3: The heatmap of lncRNAs appeared in module 1 and their mRNA neighbors based on their expression. The columns represented 6 samples and the rows represented lncRNAs and their mRNA neighbors.

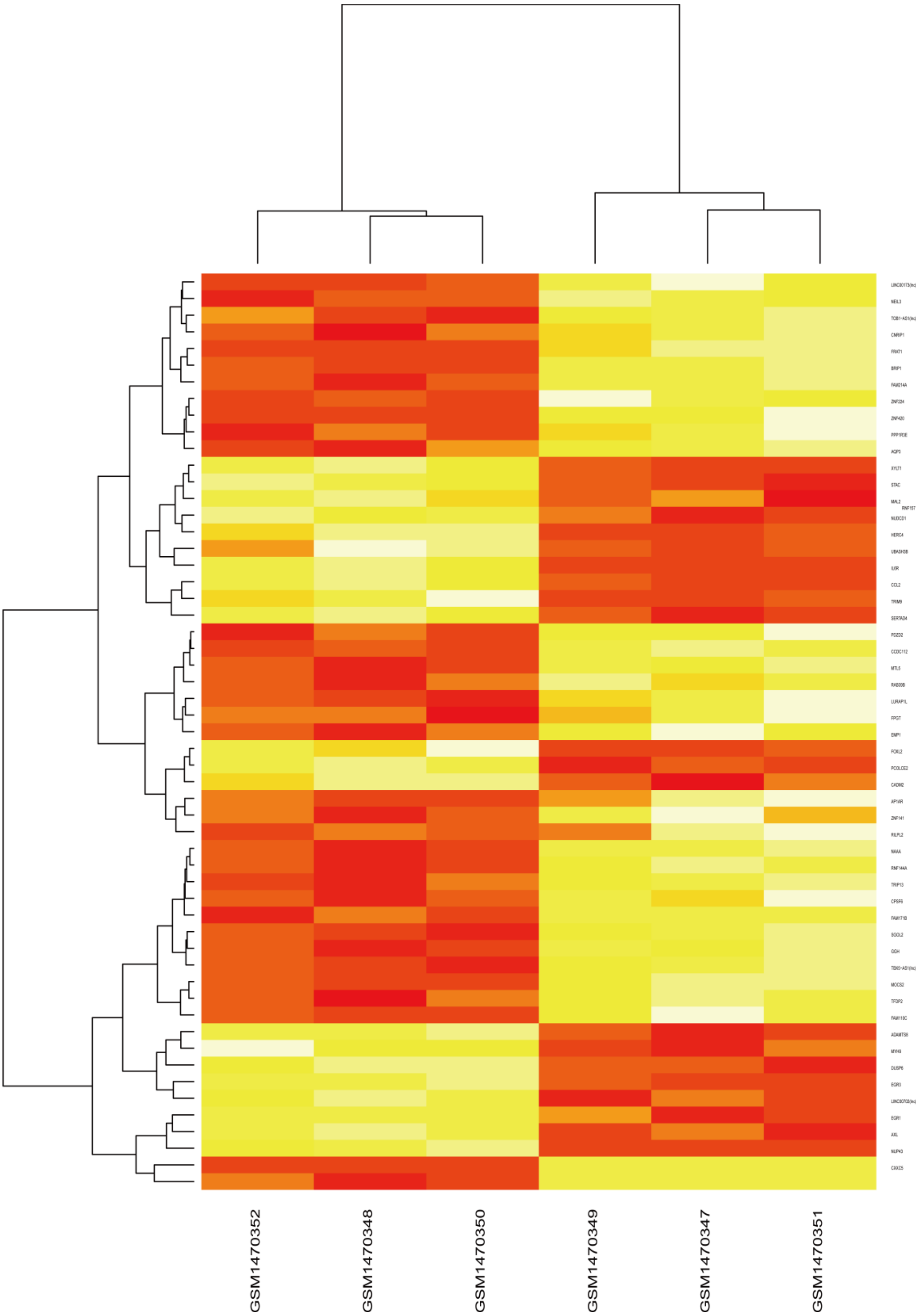

Figure S4: The heatmap of lncRNAs appeared in module 2 and their mRNA neighbors based on their expression. The columns represented 6 samples and the rows represented lncRNAs and their mRNA neighbors.
